# CCR2 Orchestrates Preferential Homing and Therapeutic Efficacy of Gingival Mesenchymal Stem Cell‐Derived Extracellular Vesicles in Rheumatoid Arthritis

**DOI:** 10.1002/mco2.70576

**Published:** 2026-01-05

**Authors:** Jingrong Chen, Xiao Guan, Wenbin Wu, Luyao Wu, Yan Liu, Donglan Zeng, Junlong Dang, Jun Zhao, Julie Wang, Jia Yuan, Xiaoli Fan, Yunfeng Pan, Nancy Olsen, Song Guo Zheng

**Affiliations:** ^1^ Division of Rheumatology Department of Medicine Songjiang Research Institute Songjiang Hospital Affiliated to Shanghai Jiao Tong University School of Medicine Shanghai China; ^2^ Department of Immunology School of Cell and Gene Therapy Songjiang Research Institute Songjiang Hospital Affiliated to Shanghai Jiao Tong University School of Medicine Shanghai China; ^3^ The State Key Laboratory of Innovative Immunotherapy at the Shanghai Jiao Tong University School of Medicine Shanghai China; ^4^ Department of Spine Surgery The Third Affiliated Hospital of Sun Yat‐Sen University Guangzhou China; ^5^ Department of Cardiology Songjiang Research Institute Songjiang Hospital Affiliated to Shanghai Jiao Tong University School of Medicine Shanghai China; ^6^ Division of Rheumatology Department of Internal Medicine The Third Affiliated Hospital of Sun Yat‐Sen University Guangzhou China; ^7^ Department of Clinical Immunology The Third Affiliated Hospital of Sun Yat‐Sen University Guangzhou China; ^8^ Department of Stomatology The Third Affiliated Hospital of Sun Yat‐Sen University Guangzhou China; ^9^ Division of Rheumatology, Department of Medicine Penn State College of Medicine Hershey Pennsylvania USA

**Keywords:** C‐C chemokine receptor type 2, chemotaxis, rheumatoid arthritis, extracellular vesicles, gingiva‐derived mesenchymal stem cells, homing, inflammation

## Abstract

The clinical utility of mesenchymal stem cells (MSCs) is often limited by pulmonary entrapment and poor systemic distribution, particularly in diseases constrained by physiological barriers such as rheumatoid arthritis (RA), where joint accessibility restricts therapeutic efficacy. This study systematically compares the immunomodulatory capacity and inflammation‐targeting potential of human gingiva‐derived MSCs (GMSCs) and their extracellular vesicles (GMSC‐EVs) in vivo. Using an experimental RA model, we demonstrate that GMSC‐EVs exhibit superior tropism to inflamed joints compared to GMSCs, resulting in significantly greater amelioration of disease severity, including reduced joint swelling, bone destruction, and balanced pathogenic T‐cell responses. Mechanistically, we identify C‐C chemokine receptor type 2 (CCR2) as the critical molecular driver of this targeted homing. Genetic ablation of CCR2 via CRISPR‐Cas9/sgRNA knockdown abolishes both the joint‐specific accumulation of GMSC‐EVs and their therapeutic efficacy. These findings elucidate the molecular basis for GMSC‐EVs tropism to arthritic lesions and establish CCR2 as a pivotal target for developing precision‐engineered EVs therapies with enhanced specificity for RA treatment.

## Graphical abstract

CCR2 orchestrates preferential homing of GMSC‐derived extracellularvesicles to inflamed joints in rheumatoid arthritis.

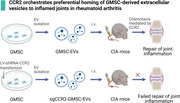

## 1 Introduction

Rheumatoid arthritis (RA) is a chronic autoimmune disorder characterized by synovial inflammation, progressive cartilage destruction, and bone erosion, leading to significant joint disability [1]. MSCs have emerged as promising therapeutic candidates for RA due to their potent immunomodulatory and tissue‐reparative capacities, primarily mediated through paracrine secretion of bioactive molecules [2, 3]. Previous studies, including our own, have demonstrated the efficacy of human GMSCs in ameliorating disease severity in various inflammatory models [4, 5, 6, 7, 8, 9, 10].

However, the clinical translation of MSC‐based therapies, including those using GMSCs, faces significant hurdles. A major limitation is the inefficient systemic distribution and poor homing of intravenously administered MSCs to the inflamed joints. This is largely attributed to their substantial entrapment within the pulmonary vasculature upon systemic delivery, drastically limiting the number of cells reaching peripheral target sites like arthritic joints [11, 12]. Consequently, achieving therapeutic concentrations at the disease site often requires high, potentially impractical, cell doses.

Extracellular vesicles (EVs) derived from MSCs have garnered increasing attention as cell‐free alternatives that recapitulate many therapeutic functions of their parent cells. MSC‐EVs carry a cargo of proteins, lipids, and nucleic acids that mirror their cellular origin and contribute to immunomodulation and tissue repair [13, 14, 15]. Crucially, their nano‐scale size confers advantages over parental MSCs, potentially enabling better penetration through biological barriers, reduced risk of pulmonary entrapment, lower immunogenicity, and enhanced safety profile [16, 17, 18].

Importantly, emerging evidence suggests that MSC‐EVs possess an intrinsic ability to selectively accumulate at sites of injury or inflammation, a property that could significantly enhance their therapeutic efficacy in diseases like RA where targeted delivery is paramount [16, 19]. While the expression of adhesion molecules (e.g., CD29, CD44, and CD73) on MSCs may facilitate tissue docking, the precise molecular mechanisms governing this preferential homing, particularly to the complex microenvironment of the inflamed joint in RA, remain incompletely understood. Chemokine receptors, known to orchestrate leukocyte trafficking in inflammation, are plausible candidates; receptors such as CCR2, CCR3, CCR4, and CXCR4 have been implicated in regulating the homing process [20, 21, 22, 23], yet their specific role in directing MSC‐EVs to arthritic lesions is unexplored.

Given the superior biodistribution potential of EVs and the critical need for targeted therapies in RA, this study aimed to (1) directly compare the therapeutic efficacy and in vivo biodistribution of human GMSCs versus GMSC‐EVs in a murine arthritis model and (2) identify the key chemokine receptor(s) responsible for the potential preferential homing of GMSC‐EVs to inflamed joints. Our findings reveal that GMSC‐EVs exhibit significantly enhanced tropism to arthritic joints compared to parental GMSCs, translating to superior therapeutic outcomes. Mechanistically, we identify CCR2 as the dominant driver of this targeted homing. Genetic ablation of CCR2 specifically within GMSC‐EVs abolished their joint‐specific accumulation and therapeutic efficacy, unequivocally establishing CCR2 as a critical molecular determinant for precision targeting in RA. These insights provide a foundation for developing CCR2‐engineered EV‐based therapeutics with enhanced specificity for RA treatment.

## 2 Results

### 2.1 Characterization and In Vitro Immunomodulatory Function of GMSCs and GMSC‐EVs

**FIGURE 1:**
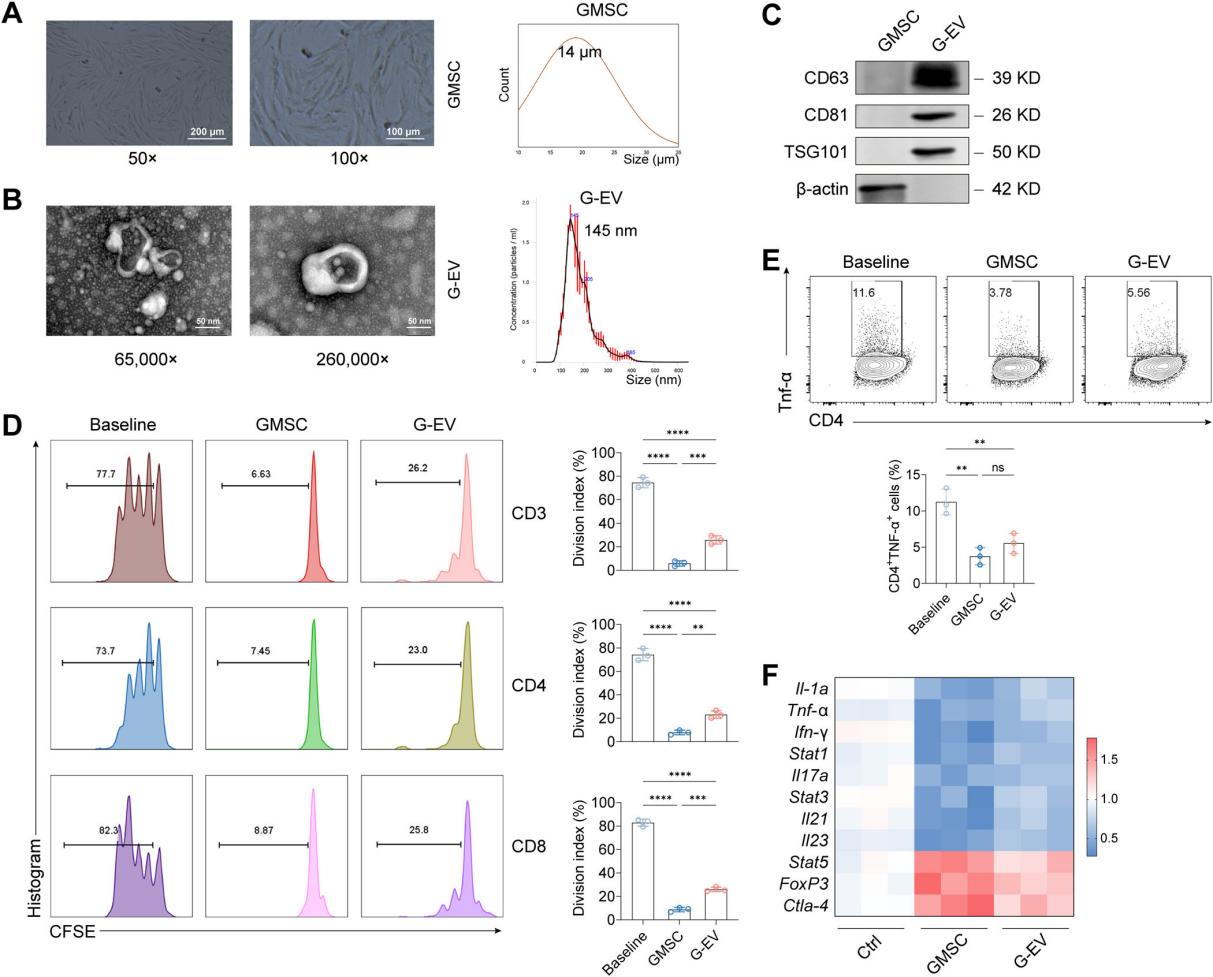
**Comparison of particle size and immunoregulatory functions between GMSCs and GMSC‐EVs**. (A) GMSC morphology and size, with scale bars at 200 and 100 µm. (B) Electron microscopy of GMSC‐EVs, with scale bars at 100 and 50 nm. (C) Western blot analysis of CD63, CD81, TSG101, and β‐actin expression in GMSCs and GMSC‐EVs. (D) In vitro assay evaluating the suppressive effect on T‐cell proliferation. (E) In vitro assay assessing TNF‐α production in CD3+ T cells. (F) qPCR analysis of relative expression levels of inflammatory and tolerogenic phenotypes in CD3+ T cells. Data are shown as the means ± SD from one of three independent experiments. ***p < 0.001; ****p < 0.0001.

GMSCs at passages three to five were isolated and expanded according to established protocols [10, 24, 25]. The typical morphology of GMSCs was captured using an electron microscope (Figure 1A, left). Microscope images were analyzed for size measurements, revealing that the majority of GMSCs ranged in size from 15 to 25 µm (Figure 1A, right). Culture medium from individual parent GMSCs was used to isolate small EVs. To confirm the identity and purity of the isolated vesicles according to minimal experimental requirements for EV characterization (MISEV guidelines), we employed transmission electron microscopy (TEM), nanoparticle tracking analysis (NTA), and immunoblotting for EV‐enriched markers. TEM revealed cup‐shaped vesicles with an approximate diameter of 140 nm (Figure 1B, left). NTA confirmed a predominant size distribution peak at 145 nm (Figure 1B, right). ​Crucially, immunoblot analysis demonstrated that GMSC‐EVs (referred to as G‐EVs or G‐EV) were enriched in canonical EV markers CD63, CD81, CD9, and TSG101, which were either absent or minimally expressed in the parental GMSCs​ (Figure 1C). ​These findings confirm the successful isolation of EVs and highlight significant differences in size and molecular phenotype between GMSCs and their derived EVs.​

​​To assess and compare the intrinsic immunomodulatory capacity of GMSCs and GMSC‐EVs, which underpins their therapeutic potential, we performed in vitro functional assays. Compared to baseline control (activated T cells without suppression),​both GMSCs and GMSC‐EVs significantly inhibited T‐cell proliferation and TNF‐α production by CD4+ T cells. However, direct comparison revealed that GMSC‐EVs exhibited a significantly potent capacity to suppress T‐cell proliferation compared to parental GMSCs (Figure 1D). In contrast, the suppressive effect of GMSC‐EVs on TNF‐α production by CD4+ T cells was comparable to that of GMSCs, as no statistically significant difference was observed between the two groups (Figure 1E). Further analysis of T‐cell activation profiles revealed that both GMSCs and GMSC‐EVs induced comparable shifts in the expression of key pro‐inflammatory or regulatory markers (Figure 1F). ​​The observed trends were consistent with the overall immunomodulatory effects demonstrated in the proliferation and cytokine suppression assays.​ Collectively, these results demonstrate that GMSC‐EVs retain significant immunomodulatory functions akin to their parental GMSCs. This functional preservation provides a rationale for investigating their therapeutic potential and biodistribution in vivo.​

### 2.2 Human GMSC‐Derived EVs Mitigate Collagen‐Induced Arthritis in an Animal Model

**FIGURE 2:**
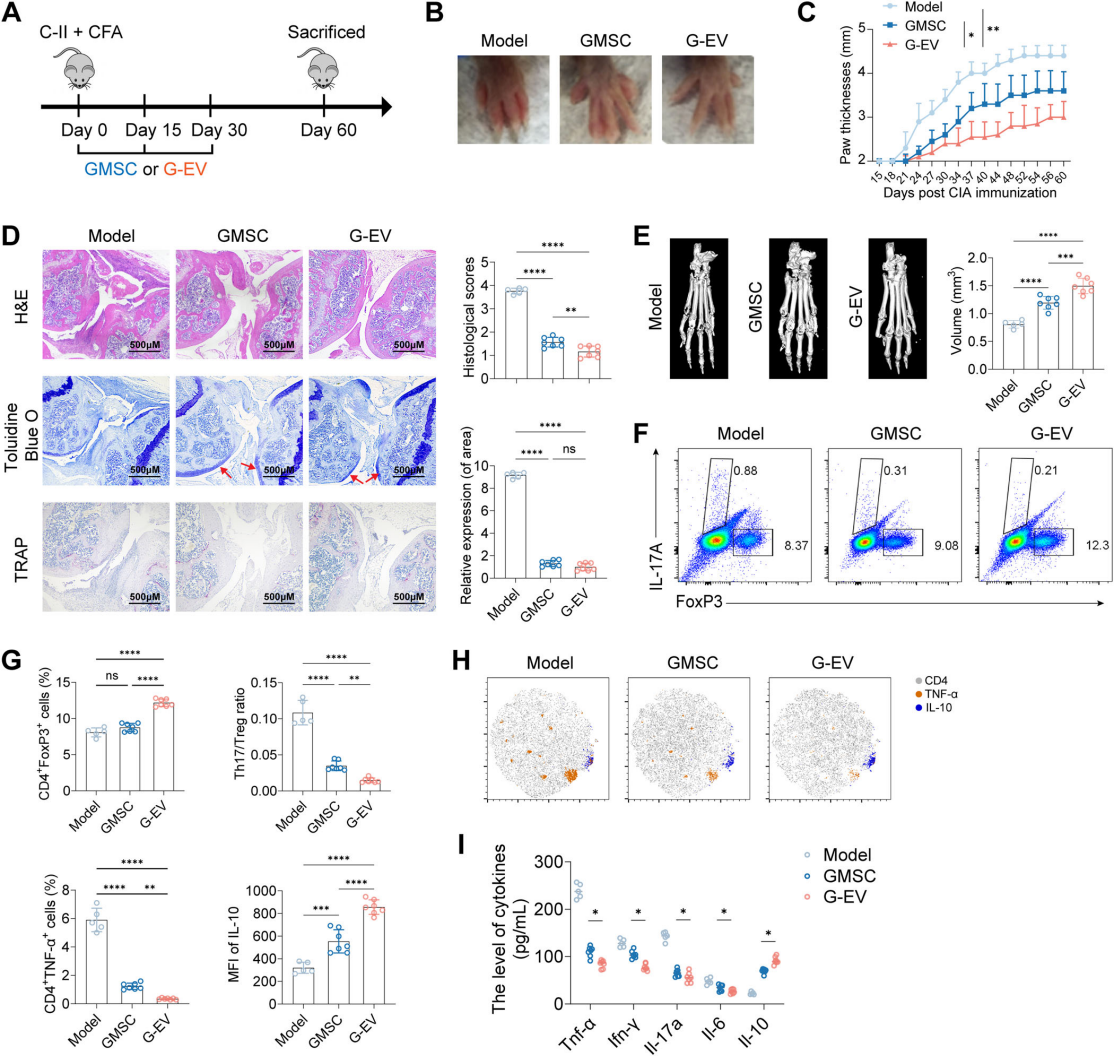
**GMSCs and GMSC‐EVs protect against collagen‐induced arthritis model**. (A) Schematic representation summarizing CIA model induction and GMSCs/GMSC‐EVs administration. (B) Representative images showing the gross appearance of swollen hind paws at the experiment's endpoint (day 60 post‐immunization). (C) Paw thickness in CIA mice was monitored from Day 15 to 60 post‐immunization. (D) Ankle joint sections from CIA mice on Day 60 were stained with H&E, TB, and TRAP. Histopathological scores were assessed for synovitis, pannus formation, erosion, and cartilage matrix destruction (indicated by red arrows). Osteoclast distribution was quantified using TRAP staining. (E) Toe joint sections from CIA mice on Day 60 were imaged with micro‐CT, and structural damage was evaluated by measuring bone volumes in the metatarsophalangeal joint. (F, G) Draining lymph node (dLN) cells from CIA mice were analyzed by flow cytometry for Il‐17a and FoxP3 expression on Day 60 post‐immunization. (H) Flow cytometry was used to assess TNF‐α and IL‐10 expression in dLN cells on Day 60 post‐immunization. (I) Serum cytokine levels (TNF‐α, IFN‐γ, Il‐17a, Il‐6, and Il‐10) were measured in CIA mice on Day 60 post‐immunization. Data are mean ± SD, *n* = 5–8 mice. *p < 0.05; **p < 0.01; ***p < 0.001; ****p < 0.0001.

To evaluate the therapeutic potential of GMSCs and their derived EVs in RA, we utilized the well‐established collagen‐induced arthritis (CIA) model in DBA/1J mice. This model recapitulates key pathological features of human RA, including synovial hyperplasia, joint swelling, and bone/cartilage destruction (Figure 2A). Treatment with GMSC‐EVs significantly attenuated the hallmark clinical signs of CIA. Specifically, GMSC‐EVs administration markedly reduced paw swelling and thickness compared to both untreated CIA controls and, notably, to treatment with parental GMSCs (Figure 2B,C). GMSC‐EVs also delayed disease onset, reduced the overall incidence of arthritis (Figure S1A), and resulted in significantly lower mean arthritis scores throughout the disease course (Figure S1B).

Consistent with the clinical improvements, histological assessment of ankle joints on Day 60 post‐immunization revealed profound protection by GMSC‐EVs. Joints from GMSC‐EVs‐treated mice exhibited substantially less inflammatory cell infiltration, synovial hyperplasia, cartilage erosion (visualized by toluidine blue [TB] staining), and bone destruction (quantified by tartrate‐resistant acid phosphatase [TRAP] staining for osteoclasts) compared to both CIA controls and GMSC‐treated mice (Figure 2D). Micro‐computed tomography (micro‐CT) analysis of metatarsophalangeal joints further confirmed the protective effect of GMSC‐EVs. Treatment with GMSC‐EVs effectively preserved bone volume and architecture, significantly mitigating the structural damage characteristic of CIA (Figure 2E).

Given the critical dysregulation of CD4+ T‐cell subsets in RA pathogenesis, we analyzed cells from draining lymph nodes (dLNs). Flow cytometry revealed that GMSC‐EVs treatment significantly modulated the balance between pathogenic Th17 cells (Il‐17a+) and regulatory T cells (FoxP3+), favoring an anti‐inflammatory state (Figure 2F,G). Furthermore, GMSC‐EVs reduced the frequency of TNF‐α‐producing CD4+ T cells while increasing the proportion of IL‐10‐producing cells within the dLN population (Figure 2H). Analysis of serum cytokine levels on day 60 demonstrated that GMSC‐EVs treatment effectively suppressed the systemic pro‐inflammatory milieu. Levels of TNF‐α, IFN‐γ, Il‐17a, and Il‐6 were significantly decreased, while the anti‐inflammatory cytokine Il‐10 was elevated (Figure 2I). ​​In addition, GMSC‐EVs treatment also significantly reduced serum levels of anti‐collagen II (CII) antibodies, including total IgG and its subclasses (IgG1, IgG2a, and IgG2b), which are key drivers of joint inflammation and destruction in CIA (Figure S1C).

Collectively, these data demonstrate that systemic administration of GMSC‐EVs provides potent therapeutic efficacy in the CIA model, significantly ameliorating both clinical and histological manifestations of arthritis. Notably, GMSC‐EVs consistently outperformed parental GMSC therapy across multiple parameters, including joint swelling reduction, disease incidence/suppression, bone preservation, and modulation of pathogenic immune responses.

### 2.3 Human GMSC‐Derived EVs Preferentially Homed Into Inflamed Joints

**FIGURE 3:**
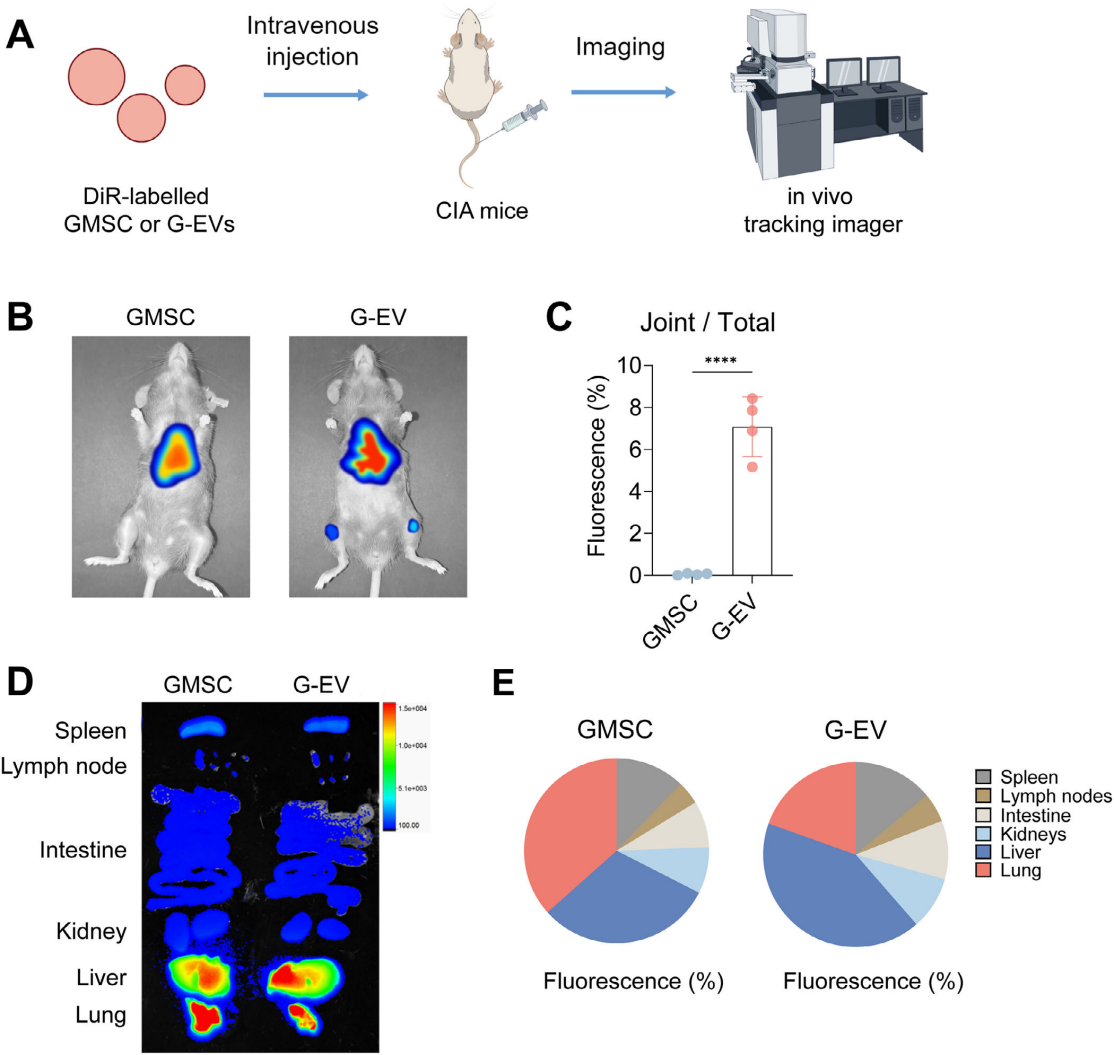
**In vivo distribution of GMSCs and GMSC‐EVs in CIA mice**. (A) Schematic illustration showing the DiR‐labeled GMSCs or GMSC‐EVs and their in vivo tracking in CIA mice. (B) Twenty‐four hours after the administration of DiR‐labeled (red) GMSCs and GMSC‐EVs in CIA mice; digital photos and IVIS images were used to display the fluorescence signals. (C) Quantification of the fluorescence percentage in joints relative to the total fluorescence observed in (B). (D, E) Similar to (B) and (C), digital photographs and IVIS images were utilized to display and quantify the fluorescence percentage in major organs, including the spleen, lymph nodes, intestines, kidneys, liver, and lungs. Data are shown as the means ± SD from one of three independent experiments. ****p < 0.0001.

To directly compare the in vivo biodistribution and inflammation‐targeting capability of GMSCs versus their derived EVs, we performed non‐invasive optical imaging in the CIA mouse model during the active phase of disease.​​ DiR, a lipophilic near‐infrared dye that incorporates into lipid membranes, was used for tracking. Mice received intravenous injections of either 2 × 10^6^ DiR‐labeled GMSCs or 100 µg DiR‐labeled GMSC‐EVs.​​ Free dye was rigorously removed from labeled GMSC‐EVs by ultracentrifugation prior to injection.​​ Whole‐body fluorescence imaging was conducted 24 h post‐injection (Figure 3A). ​​Strikingly, in vivo imaging revealed a prominent fluorescent signal specifically localized to the joints of CIA mice receiving DiR‐labeled GMSC‐EVs​​ (Figure 3B). ​​In contrast, mice injected with DiR‐labeled GMSCs exhibited minimal detectable fluorescence in the joints, with the vast majority of the signal concentrated in the lung and liver​​ (Figure 3B). ​​Quantification confirmed that the fluorescence intensity (radiance) in the joint region was significantly higher in GMSC‐EVs‐treated mice than in GMSC‐treated mice​​ (Figure 3C).

Ex vivo imaging of major organs (spleen, lymph nodes, intestine, kidney, liver, and lungs) harvested from the same mice revealed no significant differences in the distribution of fluorescence between the GMSCs and GMSC‐EVs groups​​ (Figure 3D,E). ​​This lack of difference in non‐target organs highlights the specificity of the observed accumulation of GMSC‐EVs within the inflamed arthritic joints. These results demonstrate a fundamental difference in biodistribution: while systemically administered GMSCs are predominantly trapped in the lung and liver, GMSC‐EVs efficiently bypass this entrapment and preferentially accumulate at the primary site of inflammation—the arthritic joints. This superior targeting ability to inflamed tissues likely underpins the enhanced therapeutic efficacy of GMSC‐EVs observed in the CIA model.​

### 2.4 Human GMSC‐Derived EVs Ameliorate a Xenogeneic Graft‐Versus‐Host Disease (xGvHD)

**FIGURE 4:**
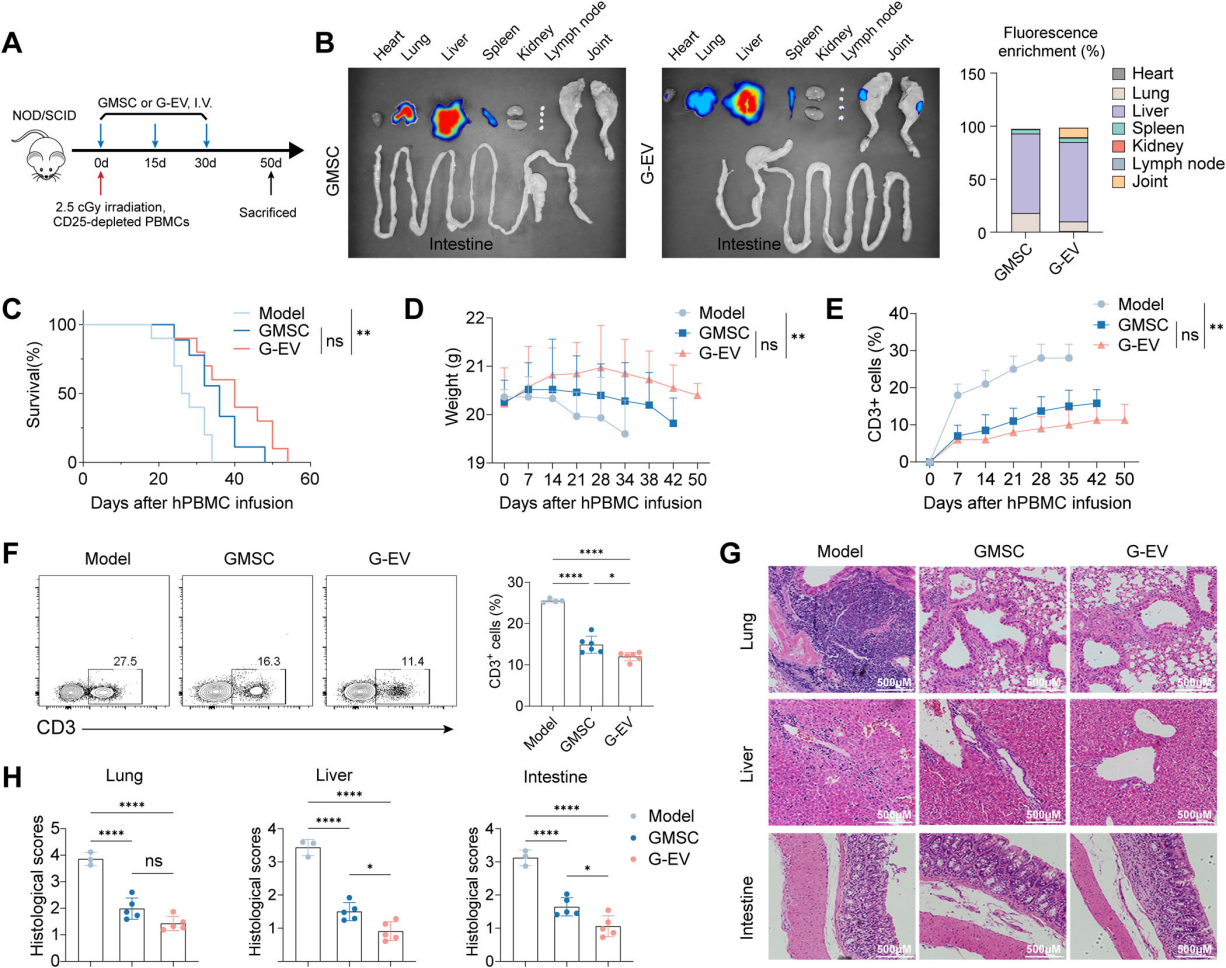
**Impact of GMSCs and GMSC‐EVs on the xGvHD model**. (A) Schematic overview of the xGvHD model experiment, including GMSCs and GMSC‐EVs administration. (B) Post‐administration of DiR‐labeled (red) GMSCs or GMSC‐EVs to xGvHD mice; digital photographs and IVIS images were used to display fluorescence signals in major organs, including the heart, lungs, liver, spleen, kidneys, lymph nodes, and joints. (C–E) xGvHD mice were treated with GMSCs or GMSC‐EVs on days 0, 15, and 30, with subsequent monitoring of survival (C), body weight (D), and human CD3+ T cell levels in peripheral blood (E). (F) Flow cytometry analysis was conducted on dLNs isolated from xGvHD mice on day 30 to determine the percentage of human CD3+ cells. (G, H) Histopathological analysis was performed on liver, lung, and intestine tissues collected from xGvHD mice on day 30, with severity scores assessed based on lymphocyte infiltration. Data are mean ± SD, *n* = 10 mice. *p < 0.05; **p < 0.01; ***p < 0.001; ****p < 0.0001.

To further investigate the hypothesis that inflamed joints present a unique physiological barrier hindering GMSC access but permitting GMSC‐EVs entry, we employed a xGvHD model. This model is characterized by robust inflammation primarily targeting barrier organs (lung, liver, and intestine), thus lacking the specific inflammatory milieu of arthritic joints.​​ Human T cells were adoptively transferred into immunodeficient NOD‐SCID mice, inducing xGvHD pathology (Figure 4A). ​​Utilizing the DiR labeling technique described previously, we tracked the biodistribution of GMSCs and GMSC‐EVs in xGvHD mice.​​

Ex vivo imaging of major organs (heart, lungs, liver, spleen, kidney, and lymph nodes) revealed a fundamental difference in biodistribution patterns. Consistent with the known phenomenon of pulmonary entrapment, DiR‐labeled GMSCs showed intense fluorescent signals predominantly in the lungs and liver. In contrast, GMSC‐EVs, owing to their nano‐scale size that minimizes mechanical trapping in the pulmonary vasculature, exhibited a more systemic distribution with reduced accumulation in these organs (Figure 4B). Crucially, and despite the absence of significant joint inflammation in this model, imaging of the joints revealed that GMSC‐EVs generated a significantly higher fluorescent signal compared to GMSCs​ (Figure 4B). This preferential accumulation of GMSC‐EVs in the joints of xGvHD mice, which lack the intense, arthritis‐specific chemokine gradient, suggests that GMSC‐EVs possess an intrinsic ability to traverse physiological barriers and home to joints. This property may be facilitated by their nanoscale size and occur via mechanisms potentially independent of, or complementary to, the classic inflammation‐driven chemokine‐receptor axis.

​​Furthermore, therapeutic administration of GMSC‐EVs on Days 0, 15, and 30 significantly ameliorated xGvHD compared to PBS controls, as evidenced by moderately improved survival rates, reduced weight loss, diminished expansion of human CD3+ T cells in peripheral blood (Figure 4C–E) and dLNs (Figure 4F), and attenuated histopathological damage in target organs (Figure 4G,H). Nevertheless, a trend toward improvement in xGvHD was observed with GMSC‐EVs treatment compared to GMSC treatment, although the difference did not reach statistical significance.​​

​​Collectively, these xGvHD findings demonstrate that GMSC‐EVs retain immunomodulatory efficacy in a systemic inflammatory disease. The superior therapeutic efficacy of GMSC‐EVs over GMSCs observed in CIA is likely dependent on their enhanced ability to overcome the joint barrier and accumulate within the arthritic lesion, a capability not required or leveraged in the xGvHD model.​

### 2.5 Profiling Identifies CCR2 as a Prominently Enriched Chemokine Receptor on Human GMSC‐Derived EVs

**FIGURE 5:**
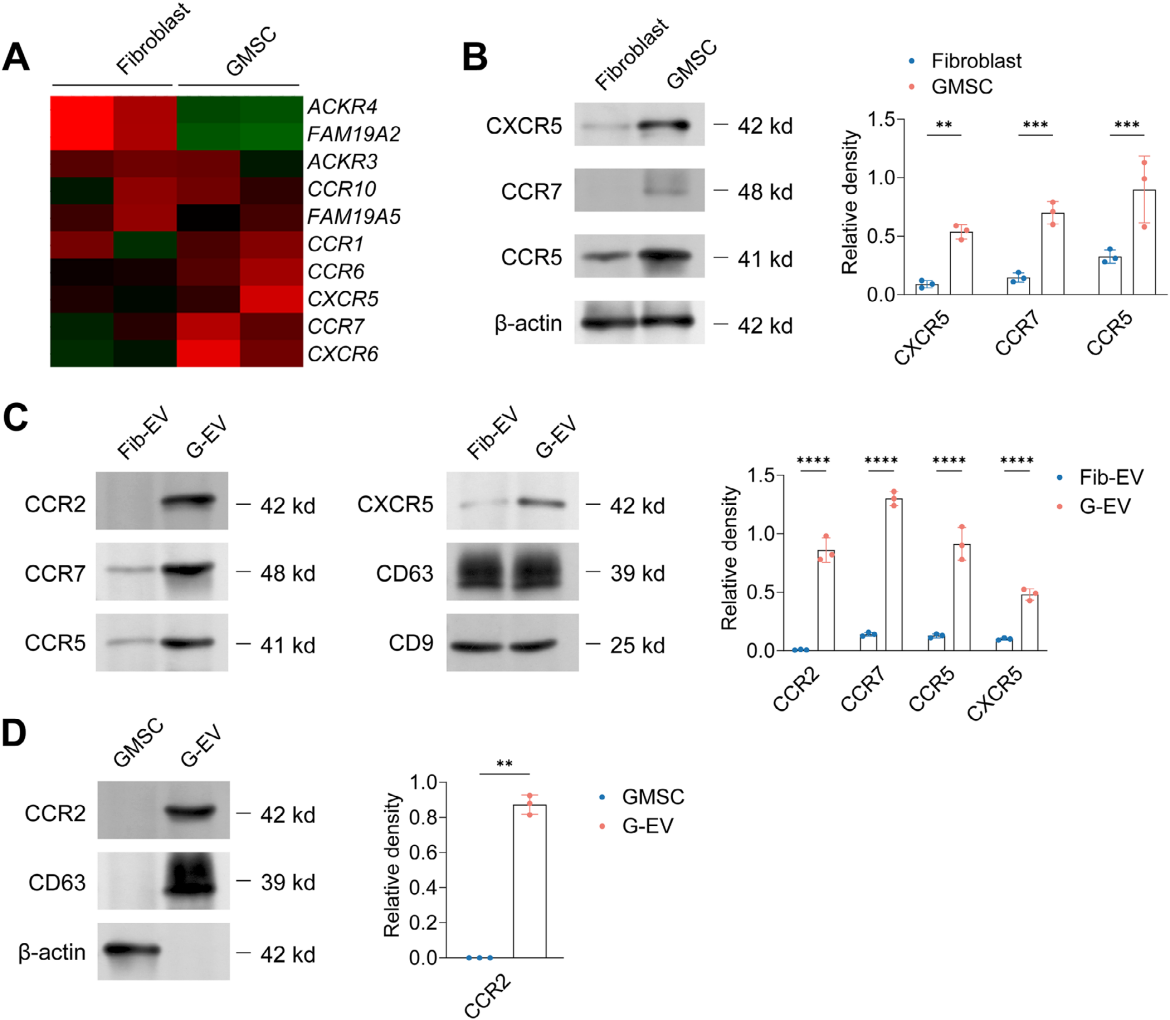
**Chemokine receptor expression patterns in GMSCs and GMSC‐EVs**. (A) Chemokine receptor family mRNA expression profile of GMSCs was presented by cluster heat map, fibroblast as the control, *p* < 0.05 was considered statistically significant. (B) The protein expression of CXCR5, CCR7, and CCR5 in GMSCs was determined by western blot, and fibroblast as the control. (C) The protein expression of CCR2, CCR7, CCR5, and CXCR5 in GMSC‐EVs was determined by western blot, and fibroblast‐EVs as the control. (D) The protein expression of CCR2 between GMSCs and GMSC‐EVs was determined by western blot. Data are shown as the means ± SD from one of three independent experiments.

Given the observed preferential accumulation of GMSC‐EVs within inflamed joints (Section 3.3), we hypothesized that chemokine receptors expressed on the EV surface might mediate this targeting by interacting with ligands abundant in the arthritic milieu. To systematically identify candidate receptors responsible for this homing, we characterized the chemokine receptor expression profiles of GMSCs and their derived GMSC‐EVs.​​ ​​First, to identify chemokine receptors associated with the MSC phenotype, we compared mRNA and protein expression profiles of GMSCs to control fibroblasts.​​ Cluster analysis of mRNA expression revealed that several receptors, including *​​CXCR6*, *CCR7*, *CXCR5*, *CCR6*, and *CCR1*, were significantly upregulated in GMSCs​​ (Figure 5A). ​​Protein analysis by Western blot confirmed significant enrichment of CXCR5, CCR7, and CCR5 in GMSCs compared to fibroblasts​​ (Figure 5B). ​​In contrast, expression levels of CCR1, CCR2, CCR3, and CCR6 did not differ significantly between GMSCs and fibroblasts​​ (Figure S2A).

​​Next, we compared the protein expression of these receptors on GMSC‐EVs to EVs derived from control fibroblasts (Fib‐EVs).​​ ​​Western blot analysis demonstrated that GMSC‐EVs were significantly enriched in CXCR5, CCR7, and CCR5 compared to Fib‐EVs​​ (Figure 5C). ​​However, expression levels of CCR1, CCR3, CCR6, CXCR4, and CXCR6 were comparable between GMSC‐EVs and Fib‐EVs​​ (Figure S2B). ​​Crucially, to pinpoint receptors potentially driving the differential targeting of GMSC‐EVs compared to their parental cells, we directly compared the expression levels of key chemokine receptors between GMSCs and GMSC‐EVs at equivalent total protein loads.​​ ​​Remarkably, while several receptors showed comparable or variable expression, CCR2 was found to be significantly enriched on GMSC‐EVs relative to GMSCs​​ (Figure 5D and Figure S2C). ​​No such enrichment was observed for CCR1, CCR3, CCR6, CXCR4, or CXCR6​​ (Figure S2C).

​​These profiling results highlight CCR2 as the most strikingly enriched chemokine receptor on GMSC‐EVs compared to their parental GMSCs. Given the pivotal role of the CCR2 ligand CCL2 in leukocyte recruitment and inflammation within the RA synovium, we posited that CCR2 expression might be a critical determinant for the targeted homing of GMSC‐EVs to arthritic joints.​​ ​​This finding provided the molecular rationale for functionally interrogating the role of CCR2 in the subsequent experiments.​

### 2.6 The Blockage of CCR2 Within Human GMSC‐Derived EVs Impairs Their Joint Targeting and Arthritic Treatment

**FIGURE 6:**
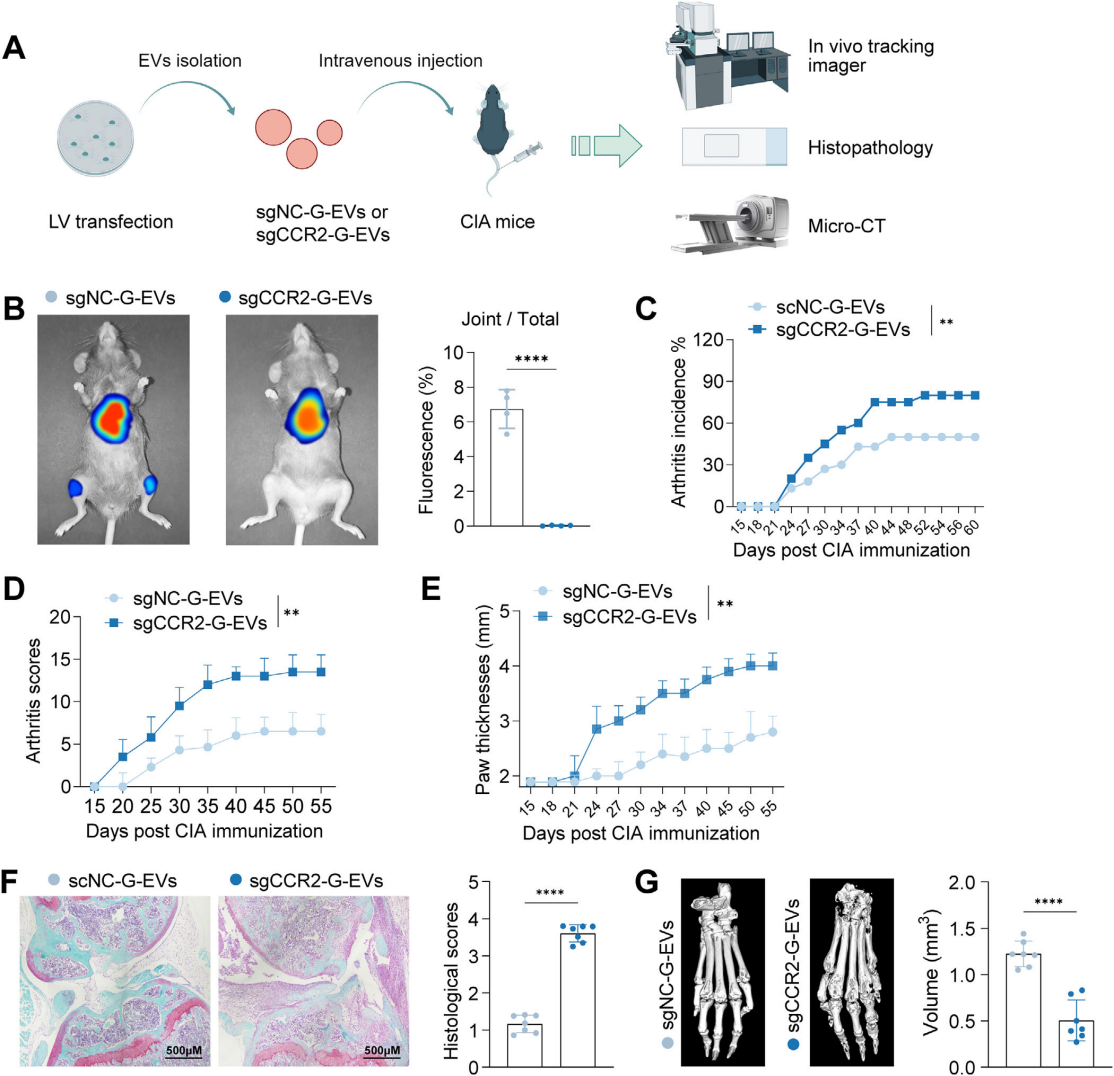
**Blocking CCR2 in GMSC‐EVs reduces their joint‐targeting ability and diminishes their therapeutic efficacy in arthritis**. (A) Schematic overview of the experimental setup for CCR2 blockade in GMSC‐EVs, including in vivo tracking, H&E staining, and micro‐CT analysis. (B) Twenty‐four hours after the administration of DiR‐labeled (red) EVs into CIA mice; digital photos and IVIS images were used to display the fluorescence signals, along with quantification of the fluorescence percentage in joints relative to total fluorescence. (C) The incidence of arthritis was tracked from Day 15 to 60 post‐immunization. (D) Arthritis severity scores were monitored from Day 15 to 60 post‐immunization. (E) Paw thickness of CIA mice was monitored from Day 15 to 60 post‐immunization. (F) Histopathologic scores of ankle joints from CIA mice on Day 60 post‐immunization were evaluated using saffron solid green staining. (G) Micro‐CT imaging of toe joint sections from CIA mice on Day 60 post‐immunization was conducted, with structural damage assessed by measuring bone volumes in the metatarsophalangeal joint. Data are mean ± SD, *n* = 5–8 mice. **p < 0.01; ****p < 0.0001.

Given the pronounced enrichment of CCR2 in GMSC‐EVs, we hypothesized that CCR2 plays a pivotal role in directing EVs to inflamed joints. To test this, we generated CCR2‐knockdown GMSCs using the CRISPR‐Cas9 system (Figure S3A,B) and followed identification of EVs from CCR2‐deficient GMSCs (referred to as sgCCR2‐G‐EVs or sgCCR2‐GMSC‐EVs) (Figure S3C). These sgCCR2‐GMSC‐EVs were administered intravenously to mice with established CIA (Figure 6A). In vivo tracking revealed that CCR2 ablation significantly impaired the homing of GMSC‐EVs to arthritic joints. Fluorescence intensity in joints was markedly reduced in sgCCR2‐GMSC‐EVs‐treated mice compared to controls injected with a non‐targeting control sgRNA (sgNC‐GMSC‐EVs or sgNC‐G‐EVs) (Figure 6B).

Crucially, this defective homing translated to diminished therapeutic efficacy. Mice receiving sgCCR2‐GMSC‐EVs exhibited higher arthritis incidence (Figure 6C), increasing average arthritis scores (Figure 6D), exacerbated paw swelling and thickness (Figure 6E), and aggravated histopathological features—including synovitis, pannus formation, and bone/cartilage erosion—relative to sgNC‐GMSC‐EVs‐treated mice (Figure 6F). Micro‐CT analysis further confirmed significantly greater bone destruction in sgCCR2‐GMSC‐EVs‐treated mice (Figure 6G). Collectively, these data establish CCR2 as a critical driver of GMSC‐EVs tropism to inflamed joints and underscore its essential role in mediating their anti‐arthritic inflammation effects.

## 3 Discussion

The therapeutic potential of MSCs for inflammatory diseases like RA is significantly hampered by their poor systemic distribution and inefficient homing to target sites [26, 27]. A major bottleneck is the profound pulmonary entrapment observed post‐systemic administration, drastically limiting the number of cells reaching peripheral inflamed tissues such as joints [11, 12]. Our study provides compelling evidence that EVs derived from human GMSC‐EVs overcome this critical limitation.

EVs have intrinsic properties that favor their accumulation in injured and inflamed tissues with abnormal vasculature rather than in normal tissues [18]. During multivesicular body formation, the EVs membrane invaginates, resulting in exosomes having the same membrane orientation as the host cell. The MSC‐EVs membrane, rich in cholesterol, sphingomyelin, ceramide, and lipid raft proteins, promotes fusion with target cells and allows trafficking through the circulatory system, overcoming biological barriers [19, 28]. Additionally, the nanoscale size of MSC‐derived EVs allows them to cross physiological barriers, leading to effective concentrations at target sites. MSC‐EVs retain their natural homing capability, aiding tissue recovery, as demonstrated in acute kidney injury models [29]. Visualizing and tracking EVs uptake by target cells in vitro and their pharmacokinetics in vivo remains technically challenging, often necessitating extensive sample purification and labeling. Lipophilic dyes such as PKH, DiO, DiI, and DiR are commonly used for labeling exosomes. Our study's initial findings reveal that GMSC‐EVs have a remarkable ability to selectively home to inflamed joints in the CIA model. This superior targeting capacity translated directly into enhanced therapeutic efficacy, as GMSC‐EVs administration resulted in significantly greater amelioration of clinical arthritis scores, paw swelling, joint inflammation, cartilage/bone destruction, and pathogenic immune responses compared to treatment with their parent GMSCs.

Notably, the yield of EVs can be influenced by the donor source of MSCs [30, 31], despite the lack of a standardized isolation method. In this study, we compared 100 µg of GMSC‐EVs, isolated using a commercial kit (requiring approximately 10 million GMSC‐derived culture supernatants), with 2 million GMSC parent cells. Results from the CIA and xGvHD animal models indicate that 100 µg of GMSC‐EVs offers superior therapeutic efficacy compared to 2 million GMSCs. Additionally, the naturally low immunogenicity of EVs ensures that allogeneic transplants are not rejected, and high‐dose exosome infusions do not induce adverse effects. Crucially, this potent therapeutic effect was achieved despite GMSC‐EVs exhibiting a reduced capacity to suppress T‐cell proliferation in vitro compared to parental GMSCs, suggesting that their in vivo efficacy is primarily driven by efficient delivery to the disease site rather than solely by maximal intrinsic immunosuppressive potency per unit dose. GMSC‐EVs’ unique biological properties, including inflammation site homing and potential for pre‐preparation, provide significant therapeutic advantages for autoimmune diseases like RA, surpassing those of GMSCs.

MSC‐EVs share their parent cells’ ability to target inflammation, and this trait remains depending on both local and systemic inflammatory environments [14]. Addressing how to optimize MSC‐EVs for enhanced homing remains crucial. Although many cell surface receptors are abundantly expressed on exosomes, their specific roles within MSC‐EVs have not been extensively examined. Chemokines and their receptors are crucial for leukocyte recruitment and angiogenesis in RA and other inflammatory rheumatic diseases. Our profiling revealed a significant enrichment of CCR2 on GMSC‐EVs compared to their parent cells at equivalent protein levels. Unlike live cells, EVs lack active migration ability. However, chemokine receptors on EVs may facilitate passive targeting through ligand‐receptor binding. Specifically, CCR2 on GMSC‐EVs may bind to cognate ligand CCL2, a chemokine abundantly expressed within the inflamed RA synovium and known to be a key driver of leukocyte recruitment and disease pathogenesis [1, 32, 33], leading to EVs retention in the joint tissue. This mechanism is analogous to the “docking” of EVs to target cells via surface receptors.​​ Importantly, genetic ablation of CCR2 via sgRNA knockdown in GMSCs and subsequent isolation of CCR2‐deficient EVs unequivocally established its pivotal role. Loss of CCR2 expression almost abolished the targeted accumulation of GMSC‐EVs in arthritic joints and completely abrogated their therapeutic benefit in CIA. This finding strongly suggests that CCR2 on GMSC‐EVs facilitates their retention or docking within the inflamed joint microenvironment. The high enrichment of CCR2 on GMSC‐EVs positions it as a critical molecular determinant for their inflammation‐targeting properties.

Beyond the inherent advantages of EVs, such as their nanoscale size facilitating barrier penetration, lower immunogenicity enabling allogeneic use, and reduced risk profile compared to live cells (e.g., avoiding uncontrolled differentiation or malignant transformation) [17, 19], our findings highlight the ​​CCR2‐dependent targeting capability​​ as a defining feature of GMSC‐EVs for RA. The enrichment of CCR2 in GMSC‐EVs (and potentially other MSC‐EVs) represents more than just a biological observation; it offers a strategic handle for therapeutic engineering. Modulating CCR2 expression levels on EVs, or leveraging CCR2‐high EVs as natural carriers, could significantly enhance the precision of drug delivery to inflamed joints. This approach holds substantial promise for improving the clinical translatability of EV‐based RA therapies by maximizing on‐target effects while minimizing systemic exposure and potential off‐target consequences.

While our study unequivocally identifies CCR2 as the dominant chemokine receptor responsible for the preferential homing of GMSC‐EVs to inflamed joints, the role of other chemokine receptors enriched on EVs, such as CCR5, CXCR4, and CXCR2, warrants further consideration. The synovial microenvironment in RA is a complex milieu characterized by the concomitant upregulation of multiple chemokines, including CCL5 (a ligand for CCR5) and CXCL12 (a ligand for CXCR4), which are critically involved in leukocyte recruitment and synovitis [32, 34, 35]. It is plausible that these receptors act in concert with CCR2 to fine‐tune the trafficking of GMSC‐EVs. For instance, the co‐expression of CCR2 and CCR5 on EVs could facilitate synergistic binding to their respective ligands, CCL2 and CCL5, which are often co‐expressed at sites of inflammation, potentially leading to more robust and specific adhesion and extravasation into the arthritic joint [35, 36]. Similarly, CXCR4, which has been implicated in the homing of MSC‐derived nanovesicles to RA joints, might contribute to the retention or deeper tissue penetration of GMSC‐EVs within the synovium [34]. Therefore, the superior homing capability of GMSC‐EVs likely results from a combinatorial effect rather than the action of a single receptor. Future studies employing multiplexed genetic approaches (e.g., simultaneous knockdown of multiple receptors) or engineered EVs with tunable receptor expression profiles are needed to dissect the potential hierarchy, cooperation, or redundancy among these chemokine receptors in orchestrating the precise targeting of EVs to inflammatory sites.

Several limitations of the current study warrant consideration. First, while the CIA model recapitulates key features of human RA, the translation of these findings to human patients requires further validation in clinical settings. Second, the optimal dosing regimen (amount and frequency) of GMSC‐EVs for sustained therapeutic efficacy in humans remains to be determined. Third, technical challenges related to the scalable production, isolation, characterization, and standardization of therapeutic‐grade EVs need to be addressed for broader clinical application [14, 37].

In conclusion, this study demonstrates that GMSC‐derived EVs possess a superior ability to home to inflamed joints compared to their parent cells, largely mediated by the enriched expression of CCR2. This intrinsic targeting mechanism underpins their enhanced therapeutic efficacy in experimental RA. Exploiting the CCR2‐CCL2 axis, either through natural EV tropism or engineered strategies, offers a promising pathway for developing more precise and effective cell‐free therapies for RA and potentially other CCR2/CCL2‐dependent inflammatory conditions.

## 4 Methods and Materials

### 4.1 Mice

This research used DBA/1 J and NOD/SCID mice obtained from Charles River Laboratories in Beijing, China. The animal experiments followed the guidelines of the Institutional Animal Care and Use Committee of the Shanghai Jiao Tong University School of Medicine, complying with both institutional and national standards for laboratory animal care and use. The study included mice ranging from 6 to 15 weeks of age. The sex of the animals was not considered a biological variable in this research. Our evaluation was focused exclusively on female mice, investigating the impact of GMSCs and GMSC‐EVs treatment in an experimental model of arthritis.

### 4.2 EVs Isolation and Identification

GMSCs were cultured in a conditioned medium with 10% EV‐depleted FBS to produce EVs. The FBS was processed through a four‐step centrifugation sequence: 300 *g* for 10 min, 3000 *g* for 10 min, 10,000 *g* for 30 minutes, and 110,000 *g* for 48 h, followed by 0.22‐µm filtration. The culture supernatant collected from this process was then used for EVs isolation utilizing Umibio EVs isolation kits (Umibio, Cat. No. UR52121, China), following established protocols [38]. Dead cells and large particles were initially removed by centrifugation at 3000 *g* for 10 min, followed by a second centrifugation at 10,000 *g* for 20 min to eliminate cell debris. The supernatant was treated with reagents according to the manufacturer's instructions, vortexed, and incubated for 2 h. EV pellets were obtained by centrifuging the mixture at 10,000 *g* for 60 min. The pellets were subsequently resuspended in 1× PBS and further purified using an EV Purification Filter with centrifugation at 3000 *g* for 10 minutes. The final EV pellet resuspension volume was 200 µL for a starting volume of 20 mL, as instructed by the manufacturer. The protein concentration of EVs was measured by BCA assay (TIANGEN, Beijing). All centrifugation steps were performed at 4°C. The isolated EVs were examined for morphology using TEM and EV markers CD63, CD81, and TSG101 were detected via western blot analysis. The particle size distribution of the EVs was measured using a NanoSight NS300 (Malvern, UK). The EVs were subsequently stored at −80°C for future analysis.

### 4.3 In Vitro Suppression Assay of T‐Cell Proliferation and Cytokine Production

To assess the inhibitory impact of GMSCs or GMSC‐EVs on T‐cell proliferation in vitro, peripheral blood mononuclear cells (PBMCs) from healthy donors were labeled with 1 µM carboxyfluorescein succinimidyl ester (CFSE). CFSE‐labeled PBMCs were cultured in 96‐well plates, stimulated with 1 µg/mL soluble anti‐CD3 and 1 µg/mL soluble anti‐CD28, and treated with GMSCs at a 1:10 ratio to T cells or GMSC‐EVs at 20 µg/mL. Following a 5‐day incubation, CD3, CD4, and CD8 T cells were analyzed via flow cytometry as previously described [39]. Additionally, for the cytokine suppression assay, TNF‐α production in CD4+ T cells was measured in healthy PBMCs (without CFSE labeling) under the same culture conditions using flow cytometry to detect TNF‐α.

### 4.4 Establishment of Collagen‐Induced RA Model

CIA model is commonly induced in genetically susceptible mice, such as DBA/1 J, by immunization with type‐II collagen (C‐II) and Freund's complete adjuvant (CFA). Following established protocols, bovine C‐II was dissolved in glacial acetic acid at 4 mg/mL. Incomplete Freund's adjuvant (IFA) was combined with heat‐inactivated *Mycobacterium tuberculosis* (6 mg/mL) to create the CFA. The mice received a 100 µL subcutaneous injection of the emulsion approximately 1.5 cm from the tail base. Based on our and other previous publications investigating the therapeutic efficacy of MSCs or MSC‐derived EVs individually [9, 40, 41, 42], in the study, each mouse received an intravenous injection of either 2 million GMSCs or 100 µg of GMSC‐EVs in 100 µL of PBS on Days 0, 15, and 30.

Arthritis symptoms were monitored every 2–3 days to assess incidence, with each mouse individually evaluated and scored for arthritis severity using previously described methods [43, 44, 45]. The scoring system was as follows: 0 points for no arthritis signs; 1 point for mild redness and swelling of the ankle or toe; 2 points for slight redness and swelling of the ankle joint, possibly extending to the toe; 3 points for moderate redness and swelling of the ankle or wrist, affecting the toe end; and 4 points for severe inflammation and swelling of the ankle joint, sole, and digit end, involving multiple joints and causing stiffness. The total arthritis score was calculated by summing the scores of all four limbs. Paw thickness was measured every 2–3 days as an indicator of joint swelling. On Day 60, mice were euthanized using CO_2_ inhalation and subsequent cervical dislocation. Hind limb knee joints were collected for histopathological analysis, evaluated on a scale from 0 (no inflammation) to 4 (severe inflammatory cell infiltration and cartilage/bone destruction) to assess synovitis, pannus formation, and bone/cartilage damage. Micro‐CT analysis was conducted on hind limb paws, calculating bone volumes of the second to fourth metatarsal and phalangeal bones based on the consistent orientation along the third metatarsal's 3D longitudinal axis. Clinical scores, arthritis incidence, paw thickness, and histological assessments were conducted by investigators blinded to the experimental conditions.

### 4.5 Establishment of a Humanized Animal Model of xGvHD

In this study, NOD‐SCID mice underwent total body irradiation at a dose of 2.5 cGy using the Rs2000 machine (Rad Source) [39, 46]. Following irradiation, 20 × 10^6^ CD25‐depleted human PBMCs were administered via intravenous injection. After 2–4 h, the mice were intravenously transfused with either 2 × 10^6^ GMSCs or 100 µg of GMSC‐EVs, both in 100 µL of PBS. Additional intravenous doses of 2 × 10^6^ GMSCs in 100 µL of PBS or 100 µg of GMSC‐EVs in 100 µL of PBS were administered on Days 15 and 30. The mice were monitored daily for survival, body weight, and GvHD scores, with weekly blood sample collections to analyze CD3+ T‐cell expansion via flow cytometry. On Day 50, the mice were euthanized via CO_2_ inhalation and cervical dislocation. Serum samples were collected for cytokine analysis, including TNF‐α, IFN‐γ, Il‐2, Il‐4, Il‐17a, and Il‐10 using ELISA kits. dLN cells were also collected for flow cytometry to determine the percentage of CD3+ T cells. Pathological examination involved hematoxylin and eosin (H&E) staining of the liver, lung, and intestine. Inflammation was scored from 0 to 4 based on the number of inflamed digits: 0 = none, 1 = 1–5, 2 = 6–10, 3 = 11–15, 4 = 16 or more.

### 4.6 Histological Evaluation

Tissues isolated from the mice were fixed in 4% paraformaldehyde and sectioned into 4‐ to 7‐µm slices. The sections were placed in a 65°C oven for 30 min to optimize tissue processing. The tissue slices were subsequently treated in two xylene baths, each for 15 min, followed by immersion in a graded series of ethanol solutions with concentrations of 100%, 95%, 85%, and 75% for 5 min each. The slices were rinsed under running water for 10 min and then stained with H&E. Cartilage matrix assessment involved TB and saffron solid green staining, while TRAP staining identified osteoclast distribution. Histological images of the stained sections were captured through photomicrography.

### 4.7 DiR Labeling and In Vivo Tracking Analysis

To monitor transplanted GMSCs or GMSC‐EVs in vivo, 2 million GMSCs or 100 µg of GMSC‐EVs were fluorescently labeled by resuspension in 1 µM DiR solution in PBS. The mixture was incubated in the dark at room temperature for 15 min. After labeling, GMSCs were rinsed with 1× PBS, while GMSC‐EVs underwent ultracentrifugation at 110,000 × *g* for 1 h. The labeled GMSCs or GMSC‐EVs were then resuspended in PBS and prepared for in vivo optical imaging. Under anesthesia, mice were intravenously injected with DiR‐labeled GMSCs or GMSC‐EVs and imaged using the Bruker in vivo MS FX PRO Imager (Bruker, Billerica, MA, USA) and the IVIS 200 small animal imaging system (PerkinElmer, Waltham, MA, USA) with 710 nm excitation and 760 nm emission. Background fluorescence was corrected by subtracting background measurements, and fluorescence emission was normalized to photons per second per centimeter squared per steradian. The spatial distribution of fluorescence was displayed in color images. Data acquisition and analysis were conducted using Living Image Software, with results presented as the mean radiance ± SD.

### 4.8 Lentivirus‐Mediated Knockdown of CCR2 in GMSC‐Derived EVs

CCR2‐knockdown GMSCs were generated using a lentivirus‐mediated CRISPR‐Cas9 system. Briefly, three specific single‐guide RNA (sgRNA) sequences targeting distinct exons of the human CCR2 gene (Gene ID: 1234) and a non‐targeting control sgRNA (sgNC) were designed, synthesized (VectorBuilder, Guangzhou), and individually cloned into the pLenti‐CRISPRv2 vector (Addgene plasmid #52961). The constructed plasmids were verified by sequencing. Lentiviral particles were produced by co‐transfecting the verified plasmids with the packaging plasmids psPAX2 and pMD2.G into HEK293T cells using a standard calcium phosphate precipitation method. The viral supernatants were collected at 48 and 72 h post‐transfection, filtered through a 0.45‐µm membrane, and concentrated by ultracentrifugation. The viral titer (transducing units/mL, TU/mL) was determined by quantitative PCR.

For infection, early‐passage human GMSCs (passage 3) were incubated with the lentiviral particles at an appropriate multiplicity of infection (MOI) in the presence of 8 µg/mL Polybrene to enhance transduction efficiency. After 18 h, the medium was replaced with a conditioned medium with 10% EV‐depleted FBS. EVs were isolated from the culture supernatants using the method described in Section 4.2. The gene editing efficiency of CCR2 protein expression in the selected sgCCR2‐GMSC‐EVs, compared to the sgNC‐GMSC‐EVs, was definitively confirmed by western blot analysis using an anti‐CCR2 antibody (Cell Signaling Technology, #12199).

### 4.9 Flow Cytometry Analysis

Surface staining for CD3, CD4, and CD8 was performed using their respective fluorescent labels. Cells were fixed and permeabilized using the FOXP3 staining buffer set (eBioscience, San Diego, CA) following the manufacturer's instructions. Cells were stimulated for 5 h with 50 ng/mL phorbol 12‐myristate 13‐acetate (PMA) and 500 ng/mL ionomycin in the presence of Brefeldin A to stain intracellular TNF‐α and Il‐17a. The cells were fixed, permeabilized, and stained following the manufacturer's guidelines. Flow cytometry data were acquired using the BD LSRFortessa (BD Biosciences, San Jose, CA, USA) and analyzed with FlowJo software version 10.6.2 (Tree Star, Ashland, OR).

### 4.10 Western Blot Analysis

Note that 30 µg of cell lysate protein was separated on a 10% SDS‐PAGE gel and transferred to PVDF membranes (Millipore, Bedford, USA). The membranes were blocked for 1 h at room temperature using 5% skim milk in TBS with 0.1% Tween 20. Following blocking, the membranes were incubated overnight at 4°C with primary antibodies against β‐actin, CCR1, CCR2, CCR3, CCR5, CCR6, CCR7, CXCR5, and CXCR6 (Abcam, UK) diluted 1:2000 in TBS with 0.1% Tween‐20. After three TBS‐T washes on a shaker, the membranes were incubated with HRP‐conjugated secondary antibodies (BOSTER, Wuhan, China) at a 1:5000 dilution for 1 h at room temperature, followed by three additional washes. Protein bands were visualized with the SuperSignal West Chemiluminescent Substrate kit (ThermoFisher, MA, USA) following the manufacturer's guidelines. The membranes were stripped and reprobed with primary and secondary antibodies to ensure equal protein loading. Autoradiographic films were scanned and analyzed with Quantity One software (Bio‐Rad, Hercules, CA).

### 4.11 Enzyme‐Linked Immunosorbent Assay

Blood samples were collected from the retro‐orbital sinus into EP tubes. The samples, without anticoagulant, were left at room temperature for 30 min to clot, then centrifuged at 12,000 *g* for 15 min. The sera were collected and stored at −80°C. TNF‐α, IFN‐γ, Il‐6, Il‐17a, Il‐10, IgG, IgG1, IgG2a, and IgG2b levels were quantified using ELISA kits (Bioo Scientific, USA) according to the manufacturer's guidelines. Each sample was analyzed in triplicate and compared to manufacturer‐provided standards, followed by statistical analysis to identify significant intergroup differences.

### 4.12 Statistical Analysis

Data are presented as mean ± SD, with all experiments conducted in triplicate. Statistical significance was evaluated using a two‐tailed Student's *t*‐test or one‐way analysis of variance (ANOVA). Survival data were illustrated using Kaplan–Meier plots and evaluated by log‐rank tests. *p*‐values below 0.05, 0.01, 0.001, and 0.0001 were deemed statistically significant. Statistical analyses were performed using GraphPad Prism Software (v9.3).

## Author Contributions

J.R.C. and X.G. conducted the experiments and analyzed the data. S.G.Z. authored the manuscript. Data collection was assisted by J.R.C., X.G., W.B.W., L.Y.W., Y.L., D.L.Z., J.L.D., and J.Z., with D.L.Z. and J.Y. specifically aiding in the collection of gingival tissues. J.R.C., X.G., and L.Y.W. revised the manuscript. Manuscript editing was supported by J.W., X.L.F., Y.F.P., and N.O. S.G.Z. conceptualized the research, designed the experiments, analyzed the data, and finalized the manuscript for submission. All authors have read and approved the final manuscript.

## Funding

This study received support from the Youth Program of National Natural Science Foundation of China (82201999, 82001760, and 82402092), the General Program of National Natural Science Foundation of China (82371817 and 82572054), the Guangdong Basic and Applied Basic Research Foundation (2025A1515012607 and 2023A1515010002), the Shanghai Songjiang District Key Science and Technology Research Project (2024SJKJGG068, 2024SJKJGG076, and 2024SJKJGG071), and the Shanghai Municipal Health Commission Collaborative Innovation Cluster Project (2024CXJQ01).

## Ethics Statement

The animal experiments followed the guidelines of the Institutional Animal Care and Use Committee of the Shanghai Jiao Tong University School of Medicine (Approval Number: No. 2024‐58), complying with both institutional and national standards for laboratory animal care and use.

## Conflicts of Interest

Song Guo Zheng is an editorial board member of *MedComm*. He was not involved in the journal's review of or decisions related to this manuscript. The other authors declare no conflicts of interest.

## Supporting information




**Figure S1. GMSCs and G‐EVs treatment in the collagen‐induced arthritis (CIA) model**. DBA/1 mice were used to establish the CIA model, receiving a single type of GMSCs or G‐EVs on days 0, 15, and 30 post‐immunization. (A) The incidence of arthritis and (B) arthritis severity scores were monitored from day 15 to day 60 post‐immunization. (C) Serum collected from CIA mice on day 60 was used to measure anti‐collagen II antibody levels via ELISA. Data are mean ± SD, n = 5‐8 mice. *, *p* < 0.05; **, *p* < 0.01.
**Figure S2. Chemokine receptor expression patterns in GMSCs and G‐EVs**. (A) Western blot analysis of CXCR6, CCR1, CCR2, CCR3, and CCR6 protein expression in GMSCs, with fibroblasts as the control. (C) Western blot analysis of CCR1, CCR3, CCR6, CXCR4, and CXCR6 protein expression in G‐EVs, using fibroblast‐derived EVs as the control. (D) Comparative Western blot analysis of CXCR6, CXCR5, CCR6, and CCR7 protein expression between GMSCs and G‐EVs. Data are shown as the means ± SD from one of three independent experiments. Data are shown as the means ± SD from one of three independent experiments.
**Figure S3. Validation of CCR2 knockout in GMSCs using CRISPR‐Cas9 and subsequent EV isolation**. (A)​ Schematic of the sgRNA‐CCR2‐CRISPR‐Cas9 plasmid construct. The sgRNA targeting sequence (sgRNA‐CCR2) is indicated. (B)​ Fluorescence imaging of reporter gene GFP expression in GMSCs following viral transduction, confirming successful infection and transduction efficiency. (C)​ Western blot analysis of CCR2 expression in EVs isolated from GMSC cultures. EVs were harvested from GMSCs transduced with either sgRNA‐CCR2 (sgCCR2‐G‐EVs) or a non‐targeting control sgRNA (sgNC‐G‐EVs). CD63 serves as a loading control. The blot confirms efficient CCR2 knockout in sgCCR2‐G‐EVs compared to control EVs. Data are shown as the means ± SD from one of three independent experiments.

## Data Availability

The data that support the findings of this study are available from the corresponding author upon reasonable request.
