# Supplementary material for: CCR2 Orchestrates Preferential Homing and Therapeutic Efficacy of Gingival Mesenchymal Stem Cell‐Derived Extracellular Vesicles in Rheumatoid Arthritis
Source: MedComm (2020). 2026 Jan 5;7(1):e70576. doi: 10.1002/mco2.70576 (PMC12771584; doi:10.1002/mco2.70576)
Supplement: Supplementary file 1 — Figure S1. GMSCs and G‐EVs treatment in the collagen‐induced arthritis (CIA) model. DBA/1 mice were used to establish the CIA model, receiving a single type of GMSCs or G‐EVs on days 0, 15, and 30 post‐immunization. (A) The incidence of arthritis and (B) arthritis severity scores were monitored from day 15 to day 60 post‐immunization. (C) Serum collected from CIA mice on day 60 was used to measure anti‐collagen II antibody levels via ELISA. Data are mean ± SD, n = 5‐8 mice. *, p < 0.05; **, p < 0.01. Figure S2. Chemokine receptor expression patterns in GMSCs and G‐EVs. (A) Western blot analysis of CXCR6, CCR1, CCR2, CCR3, and CCR6 protein expression in GMSCs, with fibroblasts as the control. (C) Western blot analysis of CCR1, CCR3, CCR6, CXCR4, and CXCR6 protein expression in G‐EVs, using fibroblast‐derived EVs as the control. (D) Comparative Western blot analysis of CXCR6, CXCR5, CCR6, and CCR7 protein expression between GMSCs and G‐EVs. Data are shown as the means ± SD from one of three independent experiments. Data are shown as the means ± SD from one of three independent experiments. Figure S3. Validation of CCR2 knockout in GMSCs using CRISPR‐Cas9 and subsequent EV isolation. (A)​ Schematic of the sgRNA‐CCR2‐CRISPR‐Cas9 plasmid construct. The sgRNA targeting sequence (sgRNA‐CCR2) is indicated. (B)​ Fluorescence imaging of reporter gene GFP expression in GMSCs following viral transduction, confirming successful infection and transduction efficiency. (C)​ Western blot analysis of CCR2 expression in EVs isolated from GMSC cultures. EVs were harvested from GMSCs transduced with either sgRNA‐CCR2 (sgCCR2‐G‐EVs) or a non‐targeting control sgRNA (sgNC‐G‐EVs). CD63 serves as a loading control. The blot confirms efficient CCR2 knockout in sgCCR2‐G‐EVs compared to control EVs. Data are shown as the means ± SD from one of three independent experiments. [file MCO2-7-e70576-s001.docx]

**Supplementary Materials**

**CCR2 Orchestrates Preferential Homing and Therapeutic Efficacy of Gingival MSC-Derived Extracellular Vesicles in Rheumatoid Arthritis**

*Jingrong Chen^1,2,3#^, Xiao Guan^1,2,3#^, Wenbin Wu^4#^, Luyao Wu^5^, Yan Liu^6^, Donglan Zeng^7^, Junlong Dang^1,2,3^, Jun Zhao^1,2,3^, Julie Wang^1,2,3^, Jia Yuan^8^, Xiaoli Fan^1*^, Yunfeng Pan^6^, Nancy Olsen^9^ and Song Guo Zheng^1,2,3*^*

^1^Division of Rheumatology, Department of Medicine, Songjiang Hospital and Songjiang Institute Affiliated to Shanghai Jiao Tong University School of Medicine, Shanghai, China

^2^Department of Immunology, School of Cell and Gene Therapy, Songjiang Research Institute, Songjiang Hospital Affiliated to Shanghai Jiao Tong University School of Medicine, Shanghai, China

^3^The State Key Laboratory of Innovative Immunotherapy at the Shanghai Jiao Tong University School of Medicine, Shanghai

^4^Department of Spine Surgery, The Third Affiliated Hospital of Sun Yat-sen University, Guangzhou, China

^5^Department of Cardiology, Songjiang Research Institute, Songjiang Hospital Affiliated to Shanghai Jiao Tong University School of Medicine, Shanghai, China

^6^Division of Rheumatology, Department of Internal Medicine, The Third Affiliated Hospital of Sun Yat-sen University, Guangzhou, China

^7^Department of Clinical Immunology, The Third Affiliated Hospital of Sun Yat-sen University, Guangzhou, China

^8^Department of stomatology, The Third Affiliated Hospital of Sun Yat-sen University, Guangzhou, China

^9^Division of Rheumatology, Department of Medicine, Penn State College of Medicine, Hershey, PA, United States

*^#^These authors contributed equally to this work.*

**Correspondence: Prof. Song Guo Zheng. Phone: +86 21 6772 2300. Email:* [*Song.Zheng@shsmu.edu.cn*](mailto:Song.Zheng@shsmu.edu.cn)*.*


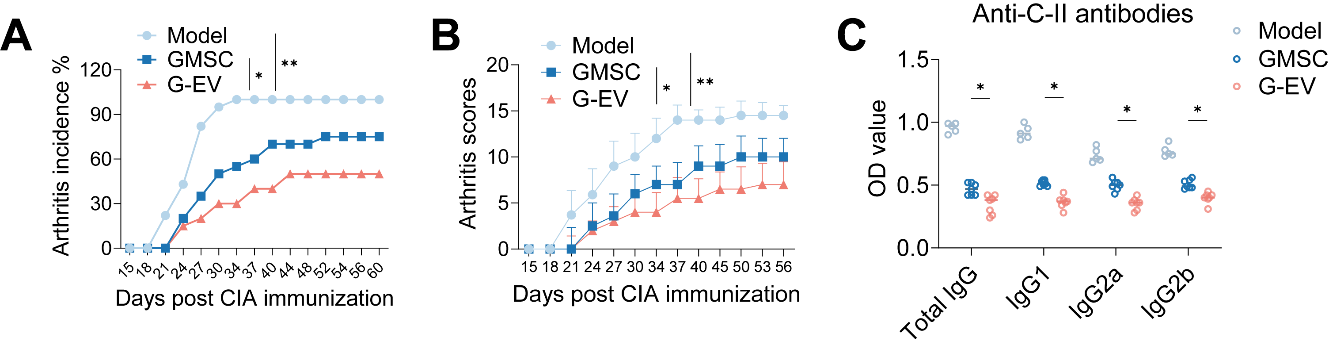


**Figure S1. GMSCs and G-EVs treatment in the collagen-induced arthritis (CIA) model.** DBA/1 mice were used to establish the CIA model, receiving a single type of GMSCs or G-EVs on days 0, 15, and 30 post-immunization. (A) The incidence of arthritis and (B) arthritis severity scores were monitored from day 15 to day 60 post-immunization. (C) Serum collected from CIA mice on day 60 was used to measure anti-collagen II antibody levels via ELISA. Data are mean ± SD, n = 5-8 mice. *, *p* < 0.05; **, *p* < 0.01.


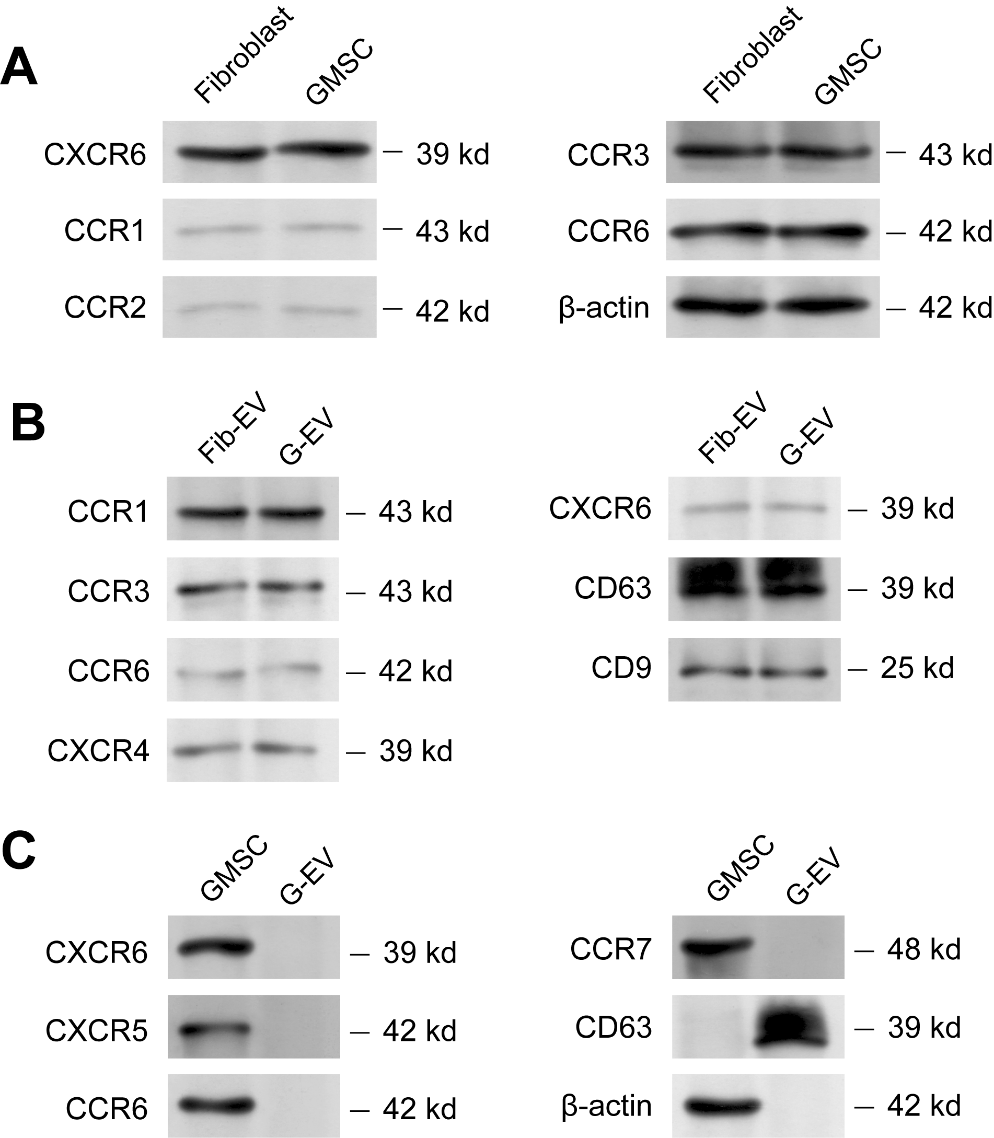


**Figure S2. Chemokine receptor expression patterns in GMSCs and G-EVs.** (A) Western blot analysis of CXCR6, CCR1, CCR2, CCR3, and CCR6 protein expression in GMSCs, with fibroblasts as the control. (C) Western blot analysis of CCR1, CCR3, CCR6, CXCR4, and CXCR6 protein expression in G-EVs, using fibroblast-derived EVs as the control. (D) Comparative Western blot analysis of CXCR6, CXCR5, CCR6, and CCR7 protein expression between GMSCs and G-EVs. Data are shown as the means ± SD from one of three independent experiments. Data are shown as the means ± SD from one of three independent experiments.


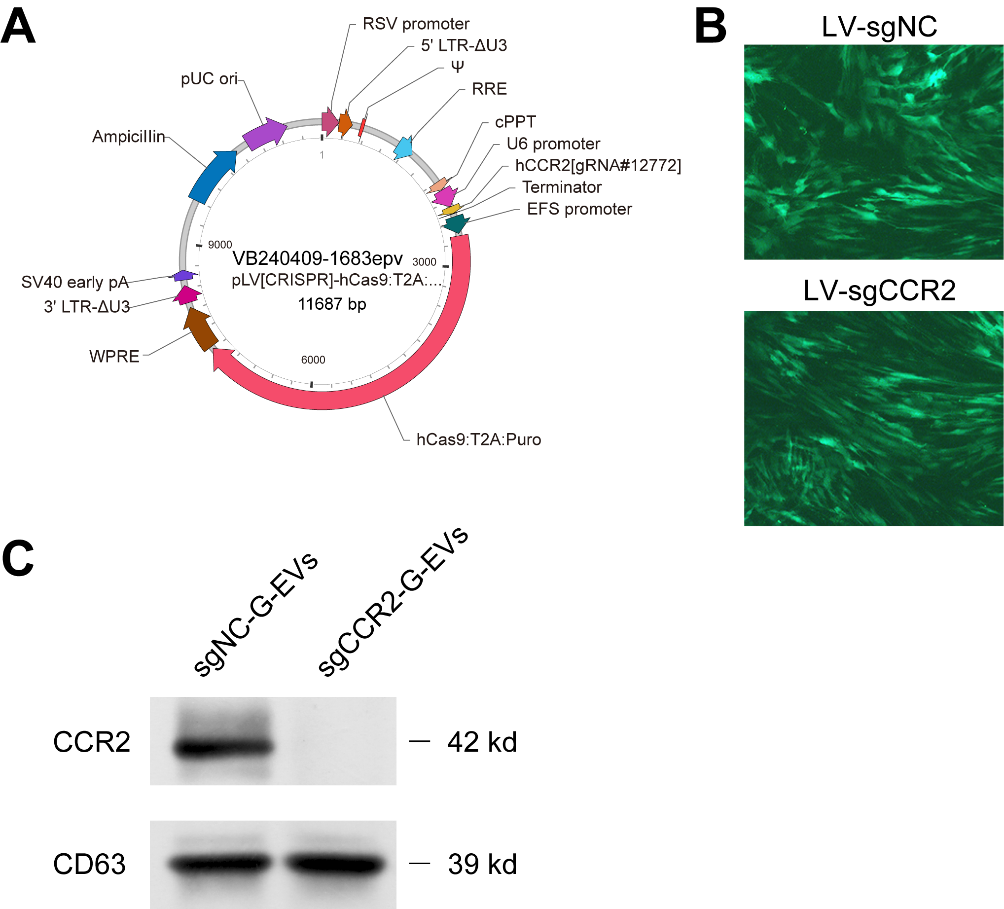


**Figure S3. Validation of CCR2 knockout in GMSCs using CRISPR-Cas9 and subsequent EV isolation.** (A)​ Schematic of the sgRNA-CCR2-CRISPR-Cas9 plasmid construct. The sgRNA targeting sequence (sgRNA-CCR2) is indicated. (B)​ Fluorescence imaging of reporter gene GFP expression in GMSCs following viral transduction, confirming successful infection and transduction efficiency. (C)​ Western blot analysis of CCR2 expression in EVs isolated from GMSC cultures. EVs were harvested from GMSCs transduced with either sgRNA-CCR2 (sgCCR2-G-EVs) or a non-targeting control sgRNA (sgNC-G-EVs). CD63 serves as a loading control. The blot confirms efficient CCR2 knockout in sgCCR2-G-EVs compared to control EVs. Data are shown as the means ± SD from one of three independent experiments.
